# Phycocyanobilin Binding and Specific Amino Acid Residues Near The Chromophore Contribute To Orange Light Perception By The Dualchrome Phytochrome Region

**DOI:** 10.1093/pcp/pcae077

**Published:** 2024-07-10

**Authors:** Mana Fukazawa, Keita Miyake, Hiroki Hoshino, Keiji Fushimi, Rei Narikawa

**Affiliations:** Department of Biological Sciences, Graduate School of Science, Tokyo Metropolitan University, 1-1 Minami-Ohsawa, Hachioji, Tokyo 192-0397, Japan; Department of General Systems Studies, Graduate School of Arts and Sciences, The University of Tokyo, 3-8-1 Komaba, Meguro, Tokyo 153-8902, Japan; Department of Biological Sciences, Graduate School of Science, Tokyo Metropolitan University, 1-1 Minami-Ohsawa, Hachioji, Tokyo 192-0397, Japan; Department of Biological Sciences, Graduate School of Science, Tokyo Metropolitan University, 1-1 Minami-Ohsawa, Hachioji, Tokyo 192-0397, Japan; Department of Biological Sciences, Graduate School of Science, Tokyo Metropolitan University, 1-1 Minami-Ohsawa, Hachioji, Tokyo 192-0397, Japan

**Keywords:** Bilin, Cyanobacteriochrome, Green alga, Phytochrome

## Abstract

A novel photoreceptor dualchrome 1 (DUC1), containing a fused structure of cryptochrome and phytochrome, was discovered in the marine green alga *Pycnococcus provasolli*. The DUC1 phytochrome region (PpDUC1-N) binds to the bilin (linear tetrapyrrole) chromophores, phytochromobilin (PΦB) or phycocyanobilin (PCB), and reversibly photoconverts between the orange-absorbing dark-adapted state and the far-red-absorbing photoproduct state. This contrasts with typical phytochromes, which photoconvert between the red-absorbing dark-adapted and far-red-absorbing photoproduct states. In this study, we examined the molecular mechanism of PpDUC1-N to sense orange light by identifying the chromophore species synthesized by *P. provasolli* and the amino acid residues within the PpDUC1-N responsible for sensing orange light in the dark-adapted state. We focused on the PcyA homolog of *P. provasolli* (PpPcyA). Coexpression with the photoreceptors followed by an enzymatic assay revealed that PpPcyA synthesized PCB. Next, we focused on the PpDUC1-N GAF domain responsible for chromophore binding and light sensing. Ten amino acid residues were selected as the mutagenesis target near the chromophore. Replacement of these residues with those conserved in typical phytochromes revealed that three mutations (F290Y/M304S/L353M) resulted in a 23-nm red shift in the dark-adapted state. Finally, we combined these constructs to obtain the PΦB-binding F290Y/M304S/L353M mutant and a 38-nm red shift was observed compared with the PCB-binding wild-type PpDUC1. The binding chromophore species and the key residues near the chromophore contribute to blue-shifted orange light sensing in the dark-adapted state of the PpDUC1-N.

## Introduction

Photosynthetic organisms contain highly organized light-response systems that acclimate to fluctuating light environments and maintain efficient photosynthesis. Terrestrial plants have developed blue and red light sensing systems to regulate various photomorphogenesis processes, such as shade avoidance, flowering and chloroplast relocation (Demarsy and Fankhauser 2009, Chen and Chory 2011, Wang et al. 2014). The phytochromes sense red light, whereas the cryptochromes and phototropins sense blue light. The phytochromes are widely distributed across the kingdoms, except for the animals and archaea (Butler et al. 1959, Hughes et al. 1997, Yeh et al. 1997, Davis et al. 1999, Blumenstein et al. 2005, Rockwell et al. 2014, Fortunato et al. 2016). They covalently bind to bilin (linear tetrapyrrole) chromophores to sense light. The Per/Arnt/Sim (PAS), cGMP phosphodiesterase/adenylate cyclase/FhlA (GAF) and phytochrome-specific (PHY) domains are required for chromophore binding and light sensing (Rockwell and Lagarias 2010, Fushimi and Narikawa 2021). Typical phytochromes exhibit reversible photoconversion between the red-absorbing, dark-adapted state binding the *Z*-isomer and the far-red-absorbing photoproduct state binding the *E*-isomer. Rotation of the double bond between the C- and D-rings of the bilin chromophore, *Z*/*E* isomerization, occurs as a primary photoreaction upon light absorption.

The phytochromes are categorized into two types based on their binding chromophore species. The plant and cyanobacterial phytochromes covalently bind to phytochromobilin (PΦB) and phycocyanobilin (PCB), which contain an ethylidene side chain of the A-ring via the canonical Cys residue within the GAF domain. In contrast, the bacterial and fungal phytochromes covalently bind to biliverdin (BV), which contains a vinyl side chain of the A-ring via the N-terminal Cys residue preceding the PAS domain. The canonical Cys residue of the plant and the cyanobacterial phytochromes bind to PΦB and PCB with no chromophore selectivity. The binding chromophore species is dependent upon ferredoxin-dependent bilin reductase (FDBR), which is encoded by the organisms. The FDBR enzymes are diverse in primary sequence and are categorized into various lineages based on varying reductions of the bilin chromophores (Sugishima et al. 2019). Terrestrial plants express HY2 enzymes to catalyze the synthesis of PΦB from BV through a one-step reduction, whereas the cyanobacteria have the PcyA enzymes to catalyze the synthesis of PCB from BV through a two-step reduction to transiently accumulate the intermediate 18^1^,18^2^-dihydrobiliverdin (18^1^,18^2^-DHBV) (Frankenberg et al. 2001, Kohchi et al. 2001). The PcyA enzyme can complement HY2 deficiency in *Arabidopsis thaliana* (Kami et al. 2004).

Recent advances in algal genomic analyses revealed the presence and characteristics of the algal phytochromes and FDBRs (Duanmu et al. 2014, 2017, Rockwell et al. 2014, 2017, Fortunato et al. 2016, Rockwell and Lagarias 2017, Frascogna et al. 2023). Algal phytochromes are highly diverse at sensing light colors compared with typical phytochromes. In particular, the unicellular green alga *Pycnococcus provasolli* has a unique photoreceptor dualchrome 1 (PpDUC1) containing a fused structure of cryptochrome and phytochrome (Makita et al. 2021). The phytochrome region of the PpDUC1 (PpDUC1-N) covalently binds to PCB or PΦB to show reversible photoconversion between the orange-absorbing, dark-adapted state and the far-red absorbing, photoproduct state. The dark-adapted state senses blue-shifted orange light compared with typical phytochromes, whose dark-adapted state senses red light.

In this study, we found that the *P. provasolli* PcyA homolog PpPcyA synthesizes PCB from BV through a two-step reduction and the key residues within the PpDUCA-N GAF domain contribute to the blue-shifted property of the dark-adapted state.

## Results

### Coexpression of PpDUC1-N and AtPhyB with PpPcyA

A multiple sequence alignment revealed that the PpPycA sequence has an N-terminal extension corresponding to a signal peptide localized to specific organelles, such as chloroplasts. PcyA enzymes localize to chloroplasts in plant cells and often have similar N-terminal signaling peptides. In previous studies, the N-terminal region of PcyA from *Chlamydomonas reinhardtii* (CrPcyA) corresponding to the signal peptide was deleted (Duanmu et al. 2013, Wittkopp et al. 2017). In the present study, we deleted the N-terminal region of PpPcyA for the coexpression and biochemical analyses. To examine the chromophore provided by PpPcyA, we constructed a vector expressing both the heme oxygenase from the model cyanobacterium *Synechocystis* sp. PCC 6803 (SyHO1) and the N-terminal-deleted PpPcyA (ΔN_PpPcyA) by replacing PcyA from *S*. 6803 of the pKT271 plasmid with ΔN_PpPcyA (Mukougawa et al. 2006). We then coexpressed His-tagged PpDUC1-N or AtPhyB with SyHO1 and ΔN_PpPcyA. The His-tagged PpDUC1-N and AtPhyB were purified by nickel-affinity chromatography and subjected to sodium dodecyl sulfate (SDS)-PAGE with Coomassie brilliant blue (CBB) staining and Zn-induced fluorescence detection (**Supplementary Fig. S1**). Zn-induced fluorescence was detected in bands at approximately 75 and 70 kDa of PpDUC1-N and AtPhyB, respectively, which were considered as the target holoproteins. The purified PpDUC1-N was nearly homogeneous as evidenced by the CBB staining, whereas the purified fraction of AtPhyB included some nonspecific proteins.

**Fig. 1:**
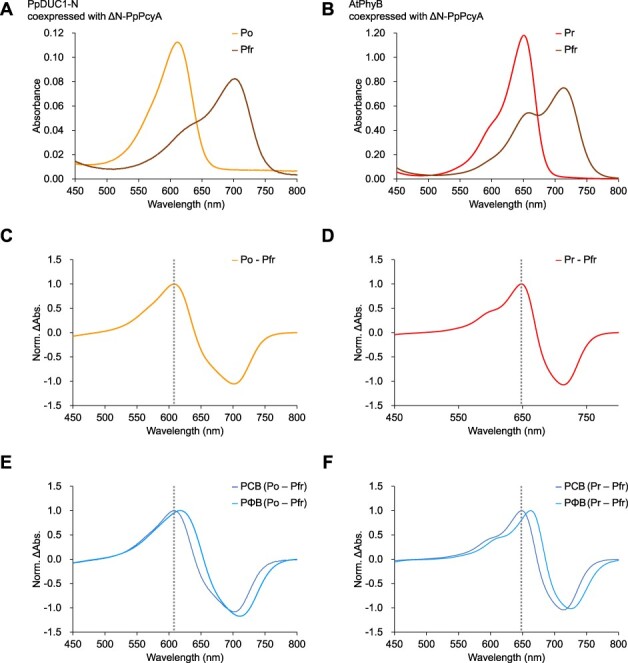
Co-expression of PpDUC1-N and AtPhyB with ΔN_PpPcyA. (A) Absorption spectra of PpDUC1-N in the Po dark-adapted and Pfr photoproduct states purified from *E. coli* co-expressing ΔN_PpPcyA. (B) Absorption spectra of AtPhyB in the Pr dark-adapted and Pfr photoproduct states purified from *E. coli* co-expressing ΔN_PpPcyA. (C) Po-minus-Pfr difference spectrum of PpDUC1-N purified from *E. coli* co-expressing ΔN_PpPcyA. (D) Pr-minus-Pfr difference spectrum of AtPhyB purified from *E. coli* co-expressing ΔN_PpPcyA. (E) Po-minus-Pfr difference spectrum of PpDUC1-N purified from PCB-producing and PΦB-producing *E. coli*. (F) Pr-minus-Pfr difference spectrum of AtPhyB purified from PCB-producing and PΦB-producing *E. coli*.

PpDUC1-N coexpressed with ΔN_PpPcyA resulted in reversible photoconversion between the orange-absorbing state (Po) peaking at 611 nm and the far-red-absorbing state (Pfr) peaking at 701 nm (**Fig. 1A**). This photoconversion was almost identical to that of PpDUC1-N purified from PCB-producing *E. coli* but different from that of PpDUC1-N purified from PΦB-producing *E. coli* (Makita et al. 2021). AtPhyB coexpressed with ΔN_PpPcyA showed reversible photoconversion between the red-absorbing state (Pr) peaking at 650 nm and the Pfr state peaking at 714 nm (**Fig. 1B**). This photoconversion was nearly identical to that of AtPhyB purified from PCB-producing *E. coli* but different from that of AtPhyB purified from PΦB-producing *E. coli*. The Po-minus-Pfr difference spectrum of PpDUC1-N coexpressed with ΔN_PpPcyA was nearly identical to that of PCB-binding PpDUC1-N, but not to that of PΦB-binding PpDUC1-N (**Fig. 1C, E**). Consistently, the Pr-minus-Pfr difference spectrum of AtPhyB coexpressed with ΔN_PpPcyA was nearly identical to that of PCB-binding AtPhyB, but not to that of the PΦB-binding AtPhyB (**Fig. 1D, F**). The acid-denatured absorption spectra of PpDUC1-N and AtPhyB coexpressed with ΔN_PpPcyA were nearly identical to those of PCB-binding PpDUC1-N, but not to those of PΦB-binding PpDUC1-N (**Supplementary Fig. S2A and B**) (Makita et al. 2021). The results strongly indicate that ΔN_PpPcyA produces PCB or chromophores like PCB.

### Enzymatic activity of ΔN_PpPcyA

**Fig. 2:**
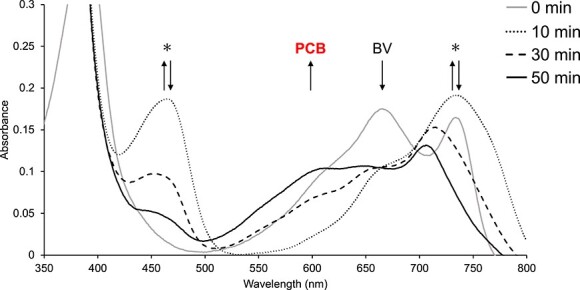
Enzymatic assay of ΔN_PpPcyA with spectroscopy. Spectroscopic observations of the reaction mixtures of ΔN_PpPcyA at various time points. Upward and downward arrows indicate an increase and decrease in absorbance, respectively. The asterisks likely correspond to radical components.

**Fig. 3:**
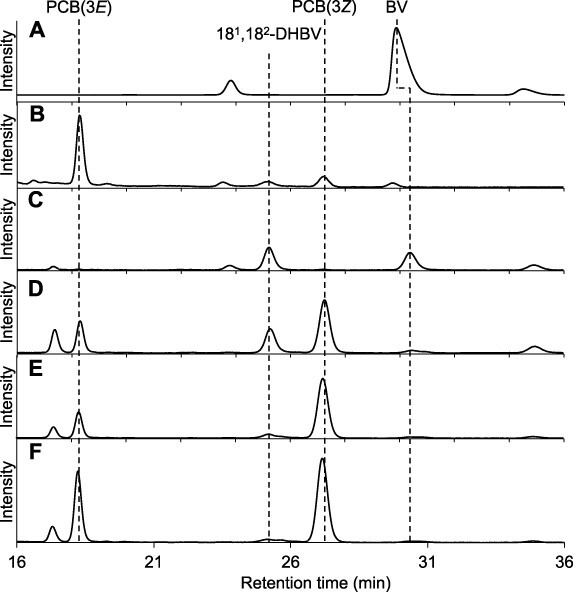
HPLC analysis of the reaction products of ΔN_PpPcyA. The chromatograms obtained at 380 nm are indicated. (A, B) BV (A) and PCB (B) as standards. (C–F) Reaction products at 10 min (C), 30 min (D), 50 min (E) and 60 min (F) after the start of the reaction.

The His-tagged ΔN_PpPcyA was purified by nickel-affinity chromatography and subjected to SDS-PAGE followed by CBB staining. A protein band at approximately 50 kDa was considered to be ΔN_PpPcyA (**Supplementary Fig. S3**). The purified ΔN_PpPcyA was analyzed by UV-vis spectroscopy and HPLC (**Figs. 2**, **3**). The enzymatic activity of ΔN_PpPcyA using BV as a substrate was monitored by spectroscopy for 60 min under anaerobic conditions (**Fig. 2**). A gradual decrease in the absorbance at 678 nm derived from BV and a concomitant increase in the absorbance at 455 and 735 nm were observed. These two peaks corresponded to the substrate radicals based on the results of previous studies (Tu et al. 2004, Miyake et al. 2020, 2022). They decreased after 10 min from the start of the enzymatic reaction, and the absorbance at 645 and 610 nm simultaneously emerged, which corresponded to the intermediate 18^1^,18^2^-DHBV and PCB, respectively, based on the previous studies (Tu et al. 2004, Miyake et al. 2020, 2022).

At several time points (0, 10, 30, 50 and 60 min) during the enzymatic reaction, we removed protein samples and extracted the chromophores for HPLC analysis (**Fig. 3**). Several peaks were detected and assigned to PCB (retention times: 17 min for the *E*-isomer and 27.8 min for the *Z*-isomer) and 18^1^,18^2^-DHBV (retention time: 25.3 min) based on the reference samples of BV and PCB and the previous studies (Miyake et al. 2020). After the enzymatic reaction was saturated (60 min), we detected small amounts of 18^1^,18^2^-DHBV, which indicated that full conversion to PCB was not achieved under the experimental conditions used in the present study (**Fig. 3F**). Taken together, ΔN_PpPcyA synthesizes PCB through the intermediate 18^1^,18^2^-DHBV from BV as the substrate as well as other PcyA enzymes.

### Mutagenesis within the GAF domain of PpDUC1-N

**Fig. 4:**
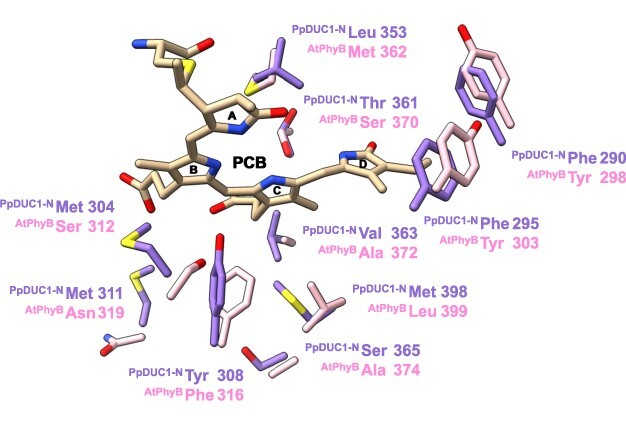
Comparison of the AtPhyB crystal structure (PDB ID: 4OUR) and the PpDUC1-N-predicted structure (AlphaFold2). Side chains of the residues different between AtPhyB and PpDUC1-N near the PCB chromophore are indicated.

The dark-adapted state of PpDUC1-N was approximately 39-nm blue-shifted compared with that of AtPhyB, even when the same chromophores were incorporated (**Fig. 1A, B**, and **Supplementary Fig. S2A and B**). This indicates that the amino acid residues around the chromophore are crucial for the blue-shifted property. The GAF domain and the tongue region within the PHY domain directly interact with the chromophore, and the PHY domain is important for the stable formation of the Pfr state, but not the Pr state in the model phytochromes (Sharda et al. 2007, Essen et al. 2008, Burgie et al. 2014). Therefore, we focused on mutating the GAF domain to identify the residues required for the blue-shifted effect. To select candidate residues, we created a structural model of PpDUC1-N and compared it with the structure of AtPhyB. We selected 10 residues around the chromophore as the mutation target, including PpDUC1-N at Phe290, Phe295, Met304, Tyr308, Met311, Leu353, Thr361, Val363, Ser365 and Met398 (**Fig. 4**). These residues were replaced with the conserved residues from AtPhyB at Tyr298, Tyr303, Ser312, Phe316, Asn319, Met362, Ser370, Ala372, Ala374 and Leu399. The variant molecules were expressed in PCB-producing *E. coli* and purified by nickel-affinity chromatography. The purified variant molecules were subjected to SDS-PAGE followed by CBB staining and Zn-induced fluorescence detection (**Supplementary Fig. S4**). Zn-induced fluorescence was detected in the 75-kDa bands, which were designated the target holoproteins. Although the purified fractions included some nonspecific proteins, proteins derived from *E. coli* did not absorb the red-light region, indicating that we could appropriately evaluate the spectral properties of the variant molecules.

**Fig. 5:**
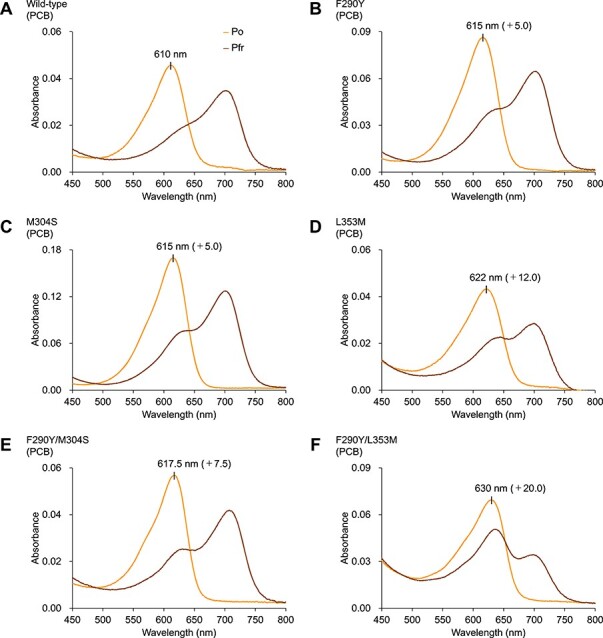
Engineering of PpDUC1-N. (A–H) Absorption spectra of the PCB-binding PpDUC1-N wild-type (A), F290Y variant (B), M304S variant (C), L353M variant (D), F290Y/M304S variant (E), F290Y/L353M variant (F), M304S/L353M variant (G) and F290Y/M304S/L353M variant (H) molecules in the Po dark-adapted and Pfr photoproduct states. (I) Normalized absorption spectra of the Po dark-adapted states of the PpDUC1-N molecules in the Po dark-adapted states (wild-type; F290Y; M304S; F290Y/M304S; L353M; F290Y/L353M; M304S/L353M; F290Y/M304S/L353M).

**Fig. 5:**
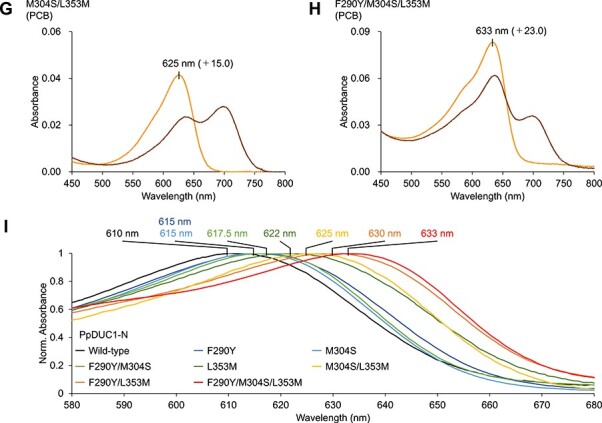
continued

We previously hypothesized that the highly twisted conformation of the D-ring would be the cause of the blue-shifted property of the dark-adapted state. Therefore, we initially focused on the residues, Phe290 and Phe295, around the D-ring (Makita et al. 2021). Single- and double-mutated variant molecules (F290Y, F295Y and F290Y/F295Y) were spectroscopically analyzed (**Supplementary Fig. S5A–D**). As a result, the dark-adapted states of the F290Y and F290Y/F295Y variant molecules exhibited a 5-nm red-shifted absorption peak at 615 nm compared with the wild-type molecule, whereas that of the F295Y variant molecule had an absorption peak at 610 nm, which was nearly identical to the wild-type molecule (**Supplementary Fig. S5A–D** and **Table S1**). The F290Y mutation, but not the F295Y mutation, contributed to the blue shift by affecting the D-ring conformation.

Next, we expanded the mutation target to the residues around the whole chromophore. To consider the synergetic effect of multiple mutations, we introduced the mutations one by one. We constructed triple-to-septuple mutant molecules (F290Y/F295Y/Y308F, F290Y/F295Y/Y308F/M304S, F290Y/F295Y/Y308F/M304S/M398L, F290Y/F295Y/Y308F/M304S/M398L/S365A and F290Y/F295Y/Y308F/M304S/M398L/S365A/V363A), in which the dark-adapted states had absorption peaks at 610 nm (no difference compared with that of the wild-type), 614.5 nm (4.5-nm red shift), 616.5 nm (6.5-nm red shift), 617.5 nm (7.5-nm red shift) and 618 nm [8.0-nm red shift, respectively (**Supplementary Fig. S5A, E–I** and **Table S1**]. By comparing the peak wavelength of a particular mutant molecule with that of its parent, mutations involved in the red-shift could be identified (**Supplementary Table S1**). For example, the M304S mutation in the quadruple mutant resulted in a 4.5-nm red shift compared with that of the triple mutant (**Supplementary Fig. S5E, F** and **Table S1**). Although further mutations were introduced to generate octuple, nonuple and decuple mutants (F290Y/F295Y/M304S/Y308F/M398L/S365A/V363A/L353M, F290Y/F295Y/M304S/Y308F/M398L/S365A/V363A/L353M/T361S and F290Y/F295Y/M304S/Y308F/M398L/S365A/V363A/L353M/T361S/M311N), these mutant molecules failed to bind to the chromophore (**Supplementary Fig. S5J–L** and **Table S1**).

The accumulation of multiple mutations should have an inhibitory effect on chromophore binding. In this context, we failed to observe the effects of the L353M, T361S and M311N mutations on the spectral properties of the dark-adapted Po state. Therefore, we constructed and compared mutant molecules (F290Y, M304S and F290Y/M304S) in which only the mutations effective for the red-shift at that time (F290Y and/or M304S) were introduced (**Fig. 5A–C and E** and **Table 1**). The dark-adapted state of the F290Y/M304S mutant exhibited an absorption peak at 617.5 nm, which was red shifted to 7.5 and 2.5 nm compared with that of the wild-type and single-mutant molecules, respectively (**Fig. 5A–C and E**, **Supplementary Fig. S6A–C and E** and **Table 1**). Based on this double mutant, we constructed triple-to-quintuple mutant molecules (F290Y/M304S/L353M, F290Y/M304S/M311N, F290Y/M304S/L353M/M311N and F290Y/M304S/L353M/M311N/T361S), whose dark-adapted states had absorption peaks at 633 nm (23-nm red shift compared with that of the wild-type), 621 nm (11-nm red shift), 634 nm (24-nm red shift) and 631 nm (21-nm red shift), respectively (**Fig. 5A, H**, **Supplementary Fig. S6A, H–K**, **Table 1** and **Supplementary Table S2**). The L353M mutation in the triple mutant resulted in a 15.5-nm red shift compared with the F290Y/M304S double mutant (**Fig. 5E, H**, **Supplementary Fig. S6E and H** and **Table 1**). A chromophore incorporated into the triple-mutant molecule was identified as PCB based on its acid-denatured absorption spectra (**Supplementary Fig. S2C**). Taken together, three mutations (F290Y, M304S and L353M) contributed to the red-shift of the dark-adapted states. Finally, to evaluate the contribution of each mutation, we constructed the L353M, F290Y/L353M and M304S/L353M mutant molecules, whose dark-adapted states had absorption peaks at 622 nm (12-nm red shift compared with that of the wild-type), 630 nm (15-nm red shift compared with that of the F290Y mutant molecule) and 625 nm (10-nm red shift compared with that of the M304S mutant molecule), respectively (**Fig. 5A–D, F and G**, **Supplementary Fig. S6A–D, F and G** and **Table 1**). We did not observe any significant synergetic effects, only additive effects, on the red shift of the dark-adapted state (**Fig. 5I**, and **Table 1**).

**Table 1:** Absorption peak shifts of the PpDUC1-N molecule in the dark-adapted states by the F290Y, M304S and L353M mutations

PpDUC1-N	λ_max_ in the dark-adapted state (nm)	Peak difference compared to wild-type (nm)
Wild-type	610.0	–
F290Y	615.0	+5.0
M304S	615.0	+5.0
L353M	622.0	+12.0
F290Y/M304S	617.5	+7.5
F290Y/L353M	630.0	+20.0
M304S/L353M	625.0	+15.0
F290Y/M304S/L353M	633.0	+23.0

The molecules were expressed in C41 pKT271_C0185 (*E. coli* harboring a PCB synthetic system).

**Fig. 6:**
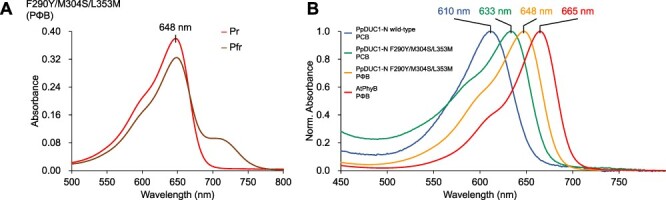
Photoconversion of the PΦB-binding F290Y/M304S/L353M variant molecule. (A) Absorption spectra of the PΦB-binding F290Y/M304S/L353M variant molecule in the Pr (red) dark-adapted and Pfr (deep red) photoproduct states. (B) Normalized absorption spectra of the PpDUC1-N and AtPhyB molecules in the dark-adapted states (PCB-binding PpDUC1-N wild-type; PCB-binding PpDUC1-N F290Y/M304S/L353M; PΦB-binding PpDUC1-N F290Y/M304S/L353M; PΦB-binding AtPhyB).

To compare with AtPhyB, which is a PΦB-binding typical phytochrome, we expressed the F290Y/M304S/L353M triple mutant in PΦB-producing *E. coli* and purified it by nickel-affinity chromatography (**Supplementary Fig. S4C**). The dark-adapted state of the PΦB-binding F290Y/M304S/L353M mutant molecule exhibited an absorption peak at 648 nm (**Fig. 6A**, and **Supplementary Fig. S2D**), which was 38- and 15-nm red shifted compared with that of the PCB-binding wild-type and triple-mutant molecules, respectively (**Fig. 5A, H**, **Fig. 6B**, **Supplementary Fig. S6A and H** and **Table 2**). This wavelength of the PΦB-binding triple-mutant molecule (648 nm) was closest to that of the PΦB-binding AtPhyB (665 nm) compared with those of the PCB-binding wild-type (610 nm) and triple mutant (633 nm) molecules (**Fig. 6B**, and **Table 2**). Overall, the specific residues, Phe290, Met304 and Leu353 in the PpDUC1-N GAF domain mainly contribute to color-tuning, namely the blue-shifted absorption in the dark-adapted state compared with those of typical phytochromes.

**Table 2:** Comparison between absorption peaks of the PpDUC1-N wild-type, F290Y/M304S/L353M mutant and AtPhyB S on the dark-adapted states

			Peak difference compared to	
Molecule	Chromophore	λ_max_ in the dark-adapted state (nm)	PCB-binding PpDUC1-N wild-type (nm)	PΦB-binding AtPhyB (nm)
PpDUC1-N wild-type	PCB	610.0	–	—55.0
PpDUC1-N F290Y/M304S/L353M	PCB	633.0	+23.0	—32.0
PpDUC1-N F290Y/M304S/L353M	PΦB	648.0	+38.0	—17.0
AtPhyB	PΦB	665.0	+55.0	–

## Discussion

In this study, we found that ΔN_PpPcyA derived from *P. provasolli* synthesized PCB from BV via 18^1^,18^2^-DHBV as well as typical PcyA enzymes (**Figs. 1**–**3**). In our previous study, we showed that BV did not act as a chromophore for PpDUC1-N (Makita et al. 2021). Furthermore, we could not find any other FDBR or BvdR homologs (Schluchter and Glazer 1997), which also function as a bilin reductase, in the *P. provasolli* genome. Based on these observations, PCB is the most plausible chromophore for the PpDUC1 phytochrome region. We demonstrated that amino acid residues within the PpDUC1-N GAF domain around the chromophore contribute to the blue-shifted, dark-adapted state (**Figs. 4**, **5**). A combination of the binding chromophore species and the amino acid residues around the chromophore enabled PpDUC1 to sense highly blue-shifted orange light with a peak at 610 nm.

The FDBR enzyme superfamily contains various lineages that reduce double bonds at different positions of the chromophore (**Supplementary Fig. S7A**). Terrestrial plants express HY2 enzymes to reduce the A-ring vinyl group of BV and to synthesize PΦB for the phytochromes (**Supplementary Fig. S7B**). Cyanobacteria contain PcyA enzymes to successively reduce the D-ring and A-ring vinyl groups of BV and to synthesize PCB, not only for the phytochromes and related photoreceptors, cyanobacteriochromes but also for the phycobiliproteins of the phycobilisome light-harvesting complex (**Supplementary Fig. S7B**). Some cyanobacteria express the other FDBR enzymes, PebA and PebB, that cooperatively and successively reduce the C15 = C16 double bond and the A-ring vinyl group to synthesize phycoerythrobilin through 15,16-DHBV for phycobiliproteins (**Supplementary Fig. S7B**). Based on a phylogenetic analysis, the HY2 and PebB enzymes are classified within the same clade (**Supplementary Fig. S7A**). These enzymes reduce the A-ring vinyl group; however, the substrates are different (BV for HY2 and 15,16-DHBV for PebB) (**Supplementary Fig. S7B**). HY2 and PebB formed a sister clade with the PebA/PebS/PubS enzymes and the PcyA enzymes are positioned as outgroup of these clades. This phylogenetic relationship is consistent with the previous studies showing that the HY2 subfamily does not originate from the PcyA subfamily but rather from the PebB subfamily (Rockwell et al. 2017, 2023, Frascogna et al. 2023).

The phylogenetic analysis of the FDBR enzymes also showed that PpPcyA belonged to the PcyA lineage (**Supplementary Fig. S7A**); however, the HY2 homologs derived from the streptophyte algae (PCB-HY2) can synthesize PCB, but not PΦB, despite belonging to the HY2 lineage (Rockwell et al. 2017, 2023, Frascogna et al. 2023). Thus, the phylogenetic analysis is not sufficient to evaluate the biochemical properties of the FDBR enzymes. Our coexpression and biochemical analyses indicated that PpPcyA is a bona fide PcyA that can synthesize PCB from BV via the intermediate 18^1^,18^2^-DHBV (**Figs. 1**–**3**). CrPcyA from the prasinophyte *C. reinhardtii* can also synthesize PCB from BV (Duanmu et al. 2013). PcyA homologs from the other prasinophytes, which form a monophyletic clade with PpPcyA and CrPcyA, can also synthesize PCB (**Supplementary Fig. S7A**). Although the green algae and plants are monophyletic, the FDBR enzymes (PcyA, PCB-HY2, PΦB-HY2 and PubS) derived from these organisms are highly diverse (**Supplementary Fig. S7A**). Green lineage organisms have lost the phycobilisome light-harvesting complex using the bilin chromophore. Furthermore, the model prasinophyte *C. reinhardtii* has further lost the phytochrome (Duanmu et al. 2013, Wittkopp et al. 2017). This may be the cause for the complex evolution of the green lineage organisms through multiple events of acquisition and loss of the FDBR enzymes. Comprehensively analyzing the distribution and biochemical properties of both the FDBRs and phytochromes will provide further insight into the evolution of the FDBRs of the green lineage organisms.

We identified three residues near the chromophore that contribute to the spectral tuning of the PpDUC1-N dark-adapted state. Of these, Leu353 is the most important for the blue shift of the dark-adapted state (**Fig. 5**). Replacement of Leu353 with Met conserved in AtPhyB resulted in a 10-nm red shift of the dark-adapted state. The Met residue in the AtPhyB is located near the A-ring (Burgie et al. 2014). In this structure, the A-ring is nearly coplanar to the B and C rings. Among the cyanobacteriochrome photoreceptors distantly related to the phytochromes, the extended red/green (XRG) cyanobacteriochromes show reversible photoconversion between the red-absorbing, dark-adapted state and the green-absorbing, photoproduct state (Narikawa et al. 2008, Chen et al. 2012, Rockwell et al. 2012). Based on the structural information, the A- and D-rings of the Pg photoproduct state are considerably twisted compared with those of the Pr dark-adapted state (Narikawa et al. 2013, Lim et al. 2018, Xu et al. 2020). The twisted geometry of these rings is considered to be the cause for the blue-shifting of the Pg photoproduct state. The branched side chain of Leu353 of PpDUC1-N may cause the A-ring configuration to become twisted, thus resulting in blue-shifting. Leu353 is highly conserved among the prasinophyte phytochromes. The A-ring twist is a common feature of the blue-shifted dark-adapted states of the prasinophyte phytochromes (Rockwell et al. 2014). Phe290 near the D-ring may contribute to the D-ring twist associated with blue-shifting. Met304 is near the B-ring propionate, which may indirectly contribute to the conformation of the A- and D-rings.

## Materials and Methods

### Bacterial strains and growth media

The *E. coli* strain JM109 was used for plasmid construction, and the *E. coli* strain C41 was used for protein expression. C41 harboring pKT271_C0185, pKT271_ΔN_PpPcyA or pKT272 was used for the co-expression experiments (Mukougawa et al. 2006, Miyake et al. 2020). pKT271_ΔN_PpPcyA was constructed as described below. *Escherichia coli* cells were grown in Luria–Bertani (LB) medium containing the appropriate antibiotics. For protein expression, the cells were grown in LB medium at 37°C until the optical density at 600 nm reached 0.4–0.8. Isopropyl-β-d-1-thiogalactopyranoside (IPTG) was added at a final concentration of 0.1 mM for the induction of protein expression. The cells were cultured at 18°C overnight, followed by the collection of the cell pellets by centrifugation.

### Plasmid construction

The gene fragment for ΔN_PpPcyA was artificially synthesized and cloned into the expression vector, pET151-D-TOPO (Thermo Fisher Scientific, Waltham, MA, USA). The ΔN_PpPcyA gene fragment was further introduced into pKT271 by replacing the SyPcyA gene fragment to generate pKT271_ΔN_PpPcyA. The plasmid expressing the PpDUC1-N was generated in a previous study (Makita et al. 2021). KOD One PCR Master Mix (TOYOBO, Osaka, Japan) was used for introducing mutations into the PpDUC1-N with the appropriate primers (**Supplementary Table S3**).

### Protein expression and purification

Proteins were expressed in *E. coli* (C41, C41 pKT271_C0185, C41 pKT271_ΔN_PpPcyA or C41 pKT272), which was incubated at 37°C until the OD_600_ ranged from 0.4 to 0.8. IPTG was added, and protein expression was induced at 18°C. The cells were collected by centrifugation at 5000 × *g* for 20 min and then frozen at −80°C for 30 min. The cells were thawed and suspended in lysis buffer [20 mM HEPES-NaOH pH7.5, 0.1 m NaCl and 10% (w/v) glycerol] and disrupted using an Emulsiflex C5 high-pressure homogenizer at 12,000 psi (Avestin, Inc., Ottawa, ON, Canada). The mixtures were centrifuged at 17,000 × *g* for 60 min. The supernatants were filtered through a 0.8-µm cellulose acetate membrane and loaded onto a nickel-affinity His-trap column (GE Healthcare, Piscataway, NJ, USA) using an ÄKTA pure system (GE Healthcare, Piscataway, NJ, USA). His-tagged proteins were purified with lysis buffer containing 100–400 mM imidazole using a linear gradient system (1 ml/min for 15 min) after washing the column with the lysis buffer containing 100 mM imidazole. EDTA (final concentration, 1 mM) was added to the purified protein, which was incubated on ice for 1 h and then dialyzed against lysis buffer to remove imidazole and EDTA. The Bradford assay (BIO-RAD Laboratories, Hercules, CA, USA) was used to measure protein concentrations. Bovine serum albumin (BSA) was used to generate a standard curve.

### Electrophoresis

The purified proteins were diluted in 60 mM dithiothreitol (DTT), 2% (w/v) SDS and 60 mM Tris-HCl, pH 8.0 for SDS-PAGE. The samples were denatured by heating at 95°C for 3 min followed by electrophoresis on 12% (w/v) acrylamide gels. The gels were soaked in 20 mM zinc acetate for 30 min to detect the fluorescence of the purified proteins. The gels were exposed to blue light (λ_max_ 470 nm) and green light (λ_max_ 527 nm) using a WSE-5500 VariRays (ATTO, Tokyo, Japan) with a short-path filter (passing through <562 nm) to visualize fluorescence from the chromophorylated proteins through a long path filter (passing through >600 nm). The fluorescent bands were imaged using a WSE109 6100 LuminoGraph (ATTO, Tokyo, Japan). After observation, the gels were stained with CBB R-250.

### Anaerobic bilin reductase assay

All solutions were de-aerated by N_2_ bubbling. The reaction mixture contained 10 μM ΔN_PpPcyA, 5 μM ferredoxin (Fd) from cyanobacterium *Synechococcus* sp. PCC 7002, 15 nM Fd NADP^+^ oxidoreductase (FNR) from cyanobacterium *Synechocystis* sp. PCC 6803, 10 μM BSA and 10 μM BV in 25 mM TES-KOH (pH 8.5). The reaction mixture was placed in a septum-stoppered quartz cuvette, and 99% pure N_2_ gas was passed for 30 min, followed by incubation at 28°C for 30 min. To control the reaction temperature while simultaneously stirring the reaction mixture, a spectrophotometer equipped with a thermoelectric single-cell holder S-1700 (SHIMADZU, Kyoto, Japan) was used. ΔN_PpPcyA activity was initiated by injecting NADPH (100 μM final concentration) into the reaction mixture into the cuvettes using a gas-tight syringe. The reaction was terminated by adding 1 ml of 0.1% trifluoroacetic acid (TFA) and incubating on ice. The products were analyzed by spectroscopy and HPLC.

### HPLC analysis

For chromophore extraction, bilin reductase assay mixtures were loaded onto Sep-Pak C18 cartridges (Waters, Milford, MA, USA) preconditioned by a 3 ml wash with acetonitrile to wet the Sep-Pak, a 3 ml wash with MilliQ water and a 3 ml wash with 0.1% (v/v) TFA. After the sample was loaded onto the Sep-Pak, it was washed with 3 ml of 0.1% (v/v) TFA and 100% acetonitrile/0.1% (v/v) TFA (20:80). The bilin products were eluted from the Sep-Pak with 1 ml of 100% acetonitrile. The eluate was passed through a 0.2-µm PTFE membrane filter and dried using a Speed-Vac evaporator. The samples were dissolved in 20 µl of dimethyl sulfoxide and then diluted with 100 µl of the HPLC mobile phase (45:55 v/v acetone: 20 mM formic acid). They were then loaded onto the HPLC system (SHIMADZU, Kyoto, Japan) which was equipped with a reverse-phase column (Kinetex C18, 2.1 i.d. × 100 mm, 1.7 μm; Phenomenex). For each sample, 10 µl aliquot was injected into the HPLC system, and absorption at 380 nm was monitored. The mobile phase consisted of acetone:20 mM formic acid [45:55 (v/v)]. The flow rate was set at 0.6 ml/min.

### Spectral analysis of the phytochromes

Ultraviolet and visible absorption spectra of the native and acid-denatured proteins were recorded using a UV-2600 spectrophotometer (SHIMADZU, Kyoto, Japan). An Opto-Spectrum Generator (Hamamatsu Photonics, Inc., Hamamatsu, Japan) was used to generate monochromic light for photoconversion. To identify the chromophores incorporated into the phytochromes, the proteins were denatured with urea under acid conditions. Details of the acid-denatured assay were reported previously (Fushimi et al. 2020).

### Structure analysis

To identify the residues responsible for the blue-shifted property, the Alphafold2-predicted structure of PpDUC1-N was constructed using the ColabFold server (Mirdita et al. 2022). This predicted structure was superimposed with the AtPhyB crystal structure (PDB ID: 4OUR). The target residues located in the GAF domains were selected near the chromophore. UCSF ChimeraX software was used for visualization and analysis of the three-dimensional structures (Pettersen et al. 2021).

### Bioinformatics

The multiple alignments of the FDBR sequences were constructed using MAFFT v7.505 (--genafpair -- maxiterate 1000) (Katoh and Standley 2013). To construct a phylogenetic tree, we used trimAl v1.4.1 (-gappyout) (Capella-Gutiérrez et al. 2009) and IQ-TREE v2.2.2.7 (-bb 1000, -alrt 1000) for maximum-likelihood (ML) phylogenetic analysis (Hoang et al. 2018, Minh et al. 2020). To select the substitution model, we used IQ-TREE’s built-in ModelFinder and selected Q.pfam + I + G4, which contained the minimized Bayesian information criterion score among 1,232 models. Statistical robustness was assessed using the ultrafast bootstrap value.

## Supplementary Material

pcae077_Supp

## Data Availability

The data underlying this study are available in the article and supplementary data.
