# Supplementary material for: Phycocyanobilin Binding and Specific Amino Acid Residues Near The Chromophore Contribute To Orange Light Perception By The Dualchrome Phytochrome Region
Source: Plant Cell Physiol. 2024 Jul 10;66(2):193–203. doi: 10.1093/pcp/pcae077 (PMC11879098; doi:10.1093/pcp/pcae077)
Supplement: pcae077_Supp [file pcae077_supp.zip › suppl_data/pcp-2024-e-00080-File007.pdf]

Table S1. Absorption peak shifts of the PpDUC1-N molecule on the dark-adapted states by introduction of the single-to-decuple mutations.

PpDUC1-N	λmax on dark-adapted state	Peak difference compared to wild-type
**Wild-type**	610.0 nm	–
**F290Y**	615.0 nm	+ 5.0 nm
**F295Y**	610.0 nm	+ 0.0 nm
**F290Y/F295Y**	615.0 nm	+ 5.0 nm
**F290Y/F295Y/Y308F**	610.0 nm	+ 0.0 nm
**F290Y/F295Y/Y308F/M304S**	614.5 nm	+ 4.5 nm
**F290Y/F295Y/Y308F/M304S/M398L**	616.5 nm	+ 6.5 nm
**F290Y/F295Y/Y308F/M304S/M398L/S365A**	617.5 nm	+ 7.5 nm
**F290Y/F295Y/Y308F/M304S/M398L/S365A/V363A**	618.0 nm	+ 8.0 nm
**F290Y/F295Y/Y308F/M304S/M398L/S365A/V363A/L353M**	Not applicable	Not applicable
**F290Y/F295Y/Y308F/M304S/M398L/S365A/V363A/L353M/T361S**	Not applicable	Not applicable
**F290Y/F295Y/Y308F/M304S/M398L/S365A/V363A/L353M/T361S/M311N**	Not applicable	Not applicable

These molecules were expressed in C41 pKT271_C0185 (*E. coli* harboring PCB synthetic system).

Table S2. Absorption peak shifts of the PpDUC1-N F290Y/M304S variant on the dark-adapted states by introduction of the L353M, M311N, and T361S mutations.

PpDUC1-N	λmax on dark-adapted state	Peak difference compared to wild-type
**Wild-type**	610.0 nm	–
**F290Y/M304S**	617.5 nm	+ 7.5 nm
**F290Y/M304S/M311N**	621.0 nm	+ 11.0 nm
**F290Y/M304S/L353M/M311N**	634.0 nm	+ 24.0 nm
**F290Y/M304S/L353M/M311N/T361S**	631.0 nm	+ 21.0 nm

These molecules were expressed in C41 pKT271_C0185 (*E. coli* harboring PCB synthetic system).

Table S3. Primer sets used in this study.

No.	Oligoname		sequence (5' -3')
1	PpPcyA_1f	Fw	GATCCCTTCACCATGCTTGGTGGTTGGAAACTGGGTC
2	PpPcyA_2r	Rv	TTTCCAACCACCAAGCATGGTGAAGGGATCAATTCCCTG
3	pKT271_PpPcyA_1f	Fw	ATTTGAGTATTTACCATGCTTGGTGGTTGGAAACTGG
4	pKT271_PpPcyA_2r	Rv	AAGCTGGAAGTGCTTTTACACATCAAACAGAACTTCGCGC
5	pKT271_PpPcyA_3f	Fw	AAGCACTTCCAGCTTTTAGCGAACATTG
6	pKT271_PpPcyA_4r	Rv	GGTAAATACTCAAATCTAGGGCAAAGGGGAATAC
7	PpDUC1_F290Y_1f	Fw	GAGCGGTATCTGAACCACTTTCCGAGCACC
8	PpDUC1_F290Y_2r	Rv	GTTCAGATACCGCTCATCTAGGTGTTCAGC
9	PpDUC1_F295Y_1f	Fw	TTACACTATCCGAGCACCGACATTCCG
10	PpDUC1_F295Y_2r	Rv	GCTCGGATAGTGTAAAACCGCTCATCTAGG
11	PpDUC1_F290Y_F295Y_1f	Fw	TTACACTATCCGAGCACCGACATTCCG
12	PpDUC1_F290Y_F295Y_2r	Rv	GCTCGGATAGTGTAATACCGCTCATCTAGG
13	PpDUC1_Y308F_1f	Fw	GCCAACTTTGTGAAAATGCGCTC
14	PpDUC1_Y308F_2r	Rv	TTTCACAAAGTTGGCACGCATCG
15	PpDUC1_Y308F_M304S_1f	Fw	AAGCGTCCCGTGCCAACTT
16	PpDUC1_Y308F_M304S_2r	Rv	CACGGGACGCTTTCGGAATGT
17	PpDUC1_M398L_1f	Fw	GGGGACTGATCGTCTGTCATCATC
18	PpDUC1_M398L_2r	Rv	GACGATCAGTCCCCACAGCAC
19	PpDUC1_S365A_1f	Fw	GTTCTGGCAATTCTGGTTCAACCG
20	PpDUC1_S365A_2r	Rv	CAGAATTGCCAGAACCAGCGTACC
21	PpDUC1_S365A_V363A_1f	Fw	GTACGCTGGCACTGGCAATTC
22	PpDUC1_S365A_V363A_2r	Rv	CCAGAATTGCCAGTGCCAGC
23	PpDUC1_T361S_1f	Fw	GGTGTGCGCGGTAGCC
24	PpDUC1_T361S_2r	Rv	GAATTGCCAGTGCCAGGCTACC
25	PpDUC1_L353M_1f	Fw	CGGTTGTCACGGCAAATATATGGAGAATATG
26	PpDUC1_L353M_2r	Rv	CGCGCACACCCATATTCTCCATATATTTG
27	PpDUC1_Y308F_M311N_1f	Fw	GCCAACTTTGTGAAGAACCGCTC
28	PpDUC1_Y308F_M311N_2r	Rv	CCCGGGAGCGGTTCTTCAC
29	PpDUC1_M304S_1f	Fw	CATTCCGAAAGCGTCCCGTGC
30	PpDUC1_M304S_2r	Rv	GTAGTTGGCACGGGACGCTTTC
31	PpDUC1_M311N_1f	Fw	CGTGCCAACTTTGTGAAAAATCGCTC
32	PpDUC1_M311N_2r	Rv	CAATGACCCGGGAGCGATTTTTCAC
33	PpDUC1_L353M_3f	Fw	GTTGTCACGGCAAATATATGGAGAATATGGG
34	PpDUC1_L353M_4r	Rv	CGCACACCCATATTCTCCATATATTTGCC
35	PpDUC1_T361S_1f	Fw	GTGCGCGGTAGCCTGGTTCTG
36	PpDUC1_T361S_2r	Rv	GCTCAGAACCAGGCTACCGCG
37	PpDUC1_S304M_1f	Fw	CATTCCGAAAGCGATGCGTGCC
38	PpDUC1_S304M_2r	Rv	CGTAGTTGGCACGCATCGCTTTC

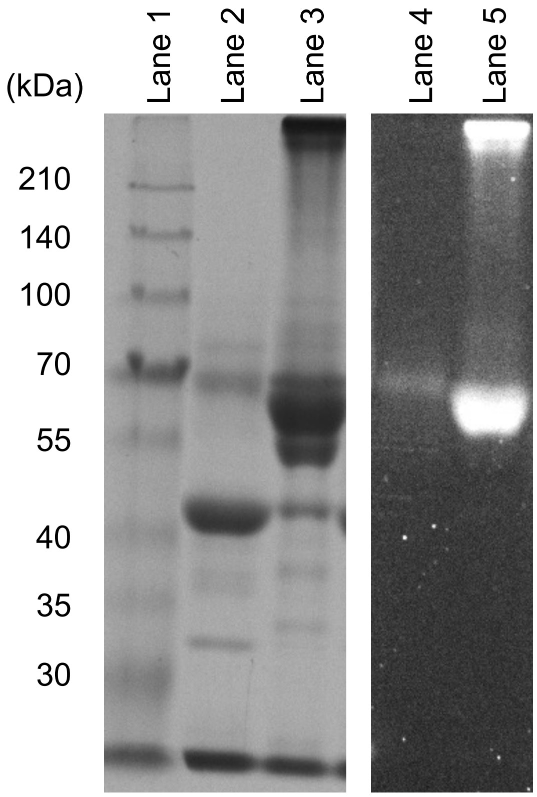

Lane 1 : Marker
Lane 2, 4 : ΔN-PpPcyA_PpDUC1-N
Lane 3, 5 : ΔN-PpPcyA_AtPhyB

Fig. S1. SDS-PAGE analysis of PpDUC1-N and AtPhyB purified from *E. coli* coexpressed with ΔN_PpPcyA. (CBB-staining: lanes 1-3, zinc-induced fluorescence: lanes 4 and 5).

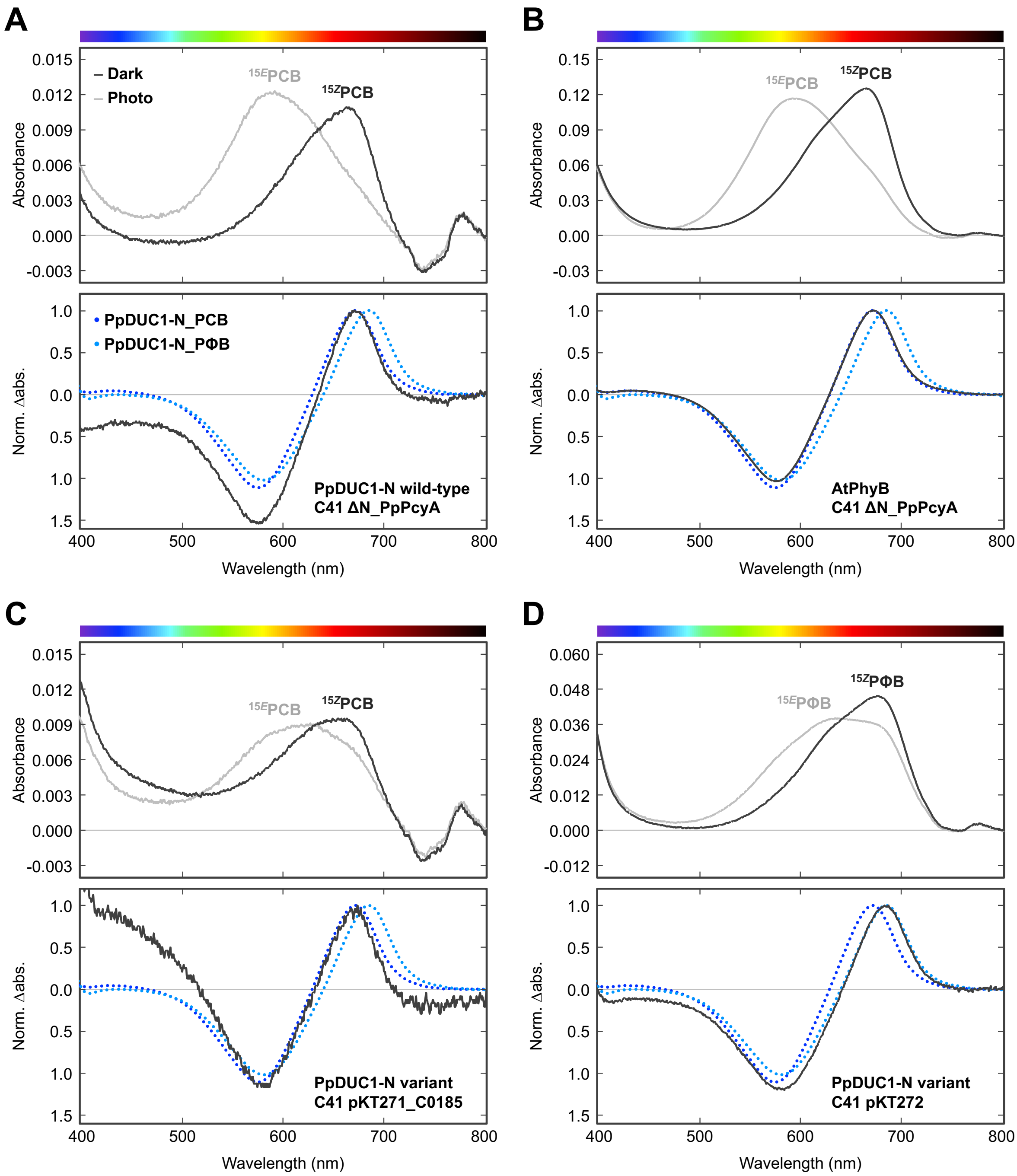

Fig. S2. Identification of chromophores incorporated into the phytochromes. (A, B) PpDUC1-N wild-type (A) and AtPhyB (B) expressed in C41 harboring ΔN_PpPcyA. (C, D) PpDUC1-N triple mutant (F290Y/M304S/L353M) molecule expressed in C41 pKT271_C0815 (C) and pKT272 (D). The normalized difference spectra (lower panels; dark-adapted state – photoproduct state) were calculated from the acid-denatured absorption spectra (upper panels; dark-adapted state (dark gray line) and photoproduct state (light gray line)). The difference spectra were compared with those of the PpDUC1-N wild-type expressed in C41 pKT271_C0815 (blue-dashed line) and C41 pKT272 (sky blue-dashed line) (Makita et al., 2021).

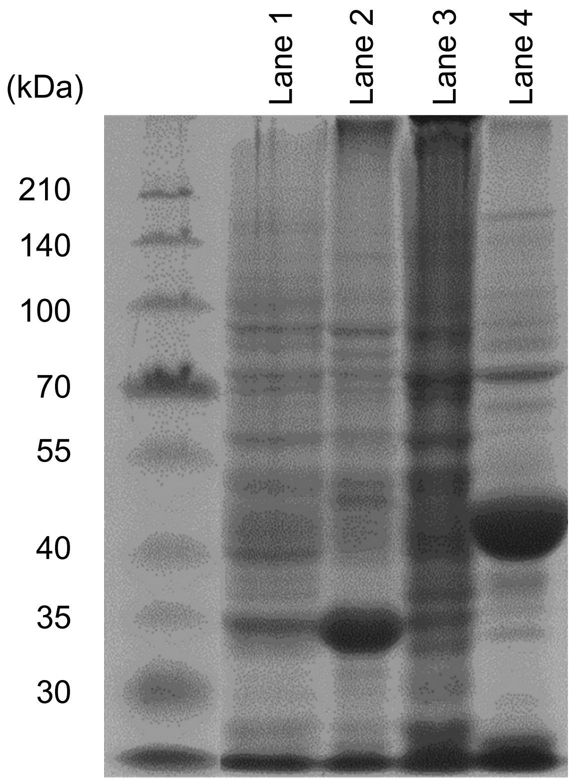

Lane1: Marker
Lane2: Pellet
Lane3: Supernatant
Lane4: Purified fraction

Fig. S3. SDS-PAGE analysis of ΔN_PpPcyA with CBB-staining.

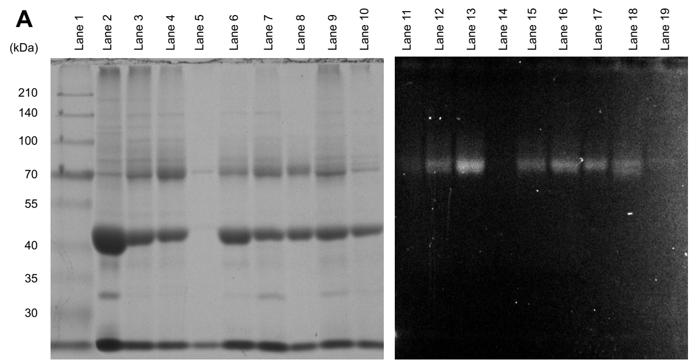

Lane 1 : Marker
Lane 2, 11 : PCB_Wild-type
Lane 3, 12 : PCB_F290Y
Lane 4, 13 : PCB_F295Y
Lane 5, 14 : PCB_F290Y/F295Y
Lane 6, 15 : PCB_F290Y/F295Y/Y308F
Lane 7, 16 : PCB_F290Y/F295Y/Y308F/M304S
Lane 8, 17 : PCB_F290Y/F295Y/Y308F/M304S/M398L
Lane 9, 18 : PCB_F290Y/F295Y/Y308F/M304S/M398L/S365A
Lane10, 19 : PCB_F290Y/F295Y/Y308F/M304S/M398L/S365A/V363A

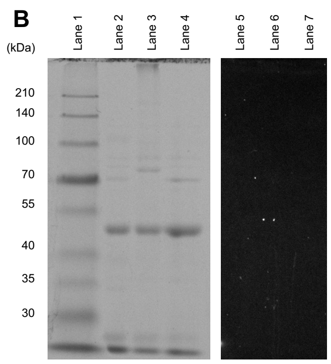

Lane 1 : Marker
Lane 2, 5 : PCB_F290Y/F295Y/Y308F/M304S/M398L/S365A/V363A/L353M
Lane 3, 6 : PCB_F290Y/F295Y/Y308F/M304S/M398L/S365A/V363A/L353M/T361S
Lane 4, 7 : PCB_F290Y/F295Y/Y308F/M304S/M398L/S365A/V363A/L353M/T361S/M311N

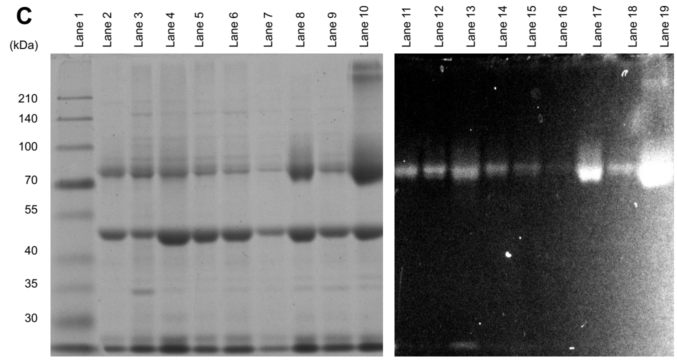

Lane 1 : Marker
Lane 2, 11 : PCB_F290Y/M304S
Lane 3, 12 : PCB_F290Y/L353M
Lane 4, 13 : PCB_F290Y/M304S/L353M
Lane 5, 14 : PCB_F290Y/M304S/M311N
Lane 6, 15 : PCB_F290Y/M304S/L353M/M311N
Lane 7, 16 : PCB_F290Y/M304S/L353M/M311N/T361S
Lane 8, 17 : PCB_M304S
Lane 9, 18 : PCB_L353M
Lane10, 19 : PΦB_F290Y/M304S/L353M

Fig. S4. SDS-PAGE analysis of the PpDUC1-N wild-type and its variant molecules. (A) Purified single-to-septuple mutant molecules expressed in C41 pKT271-C0185 (CBB-staining: lanes 1-10, zinc-induced fluorescence: lanes 11-19). (B) Purified octuple-to-decuple mutant molecules expressed in C41 pKT271-C0185 (CBB-staining: lanes 1-4, zinc-induced fluorescence: lanes 5-7). (C) Purified PpDUC1-N mutant molecules expressed in C41 pKT271-C0185 or C41 pKT272 to evaluate the contribution of F290Y, M304S, and L353M mutations to spectral-tuning (CBB-staining: lanes 1-10, zinc-induced fluorescence: lanes 11-19).

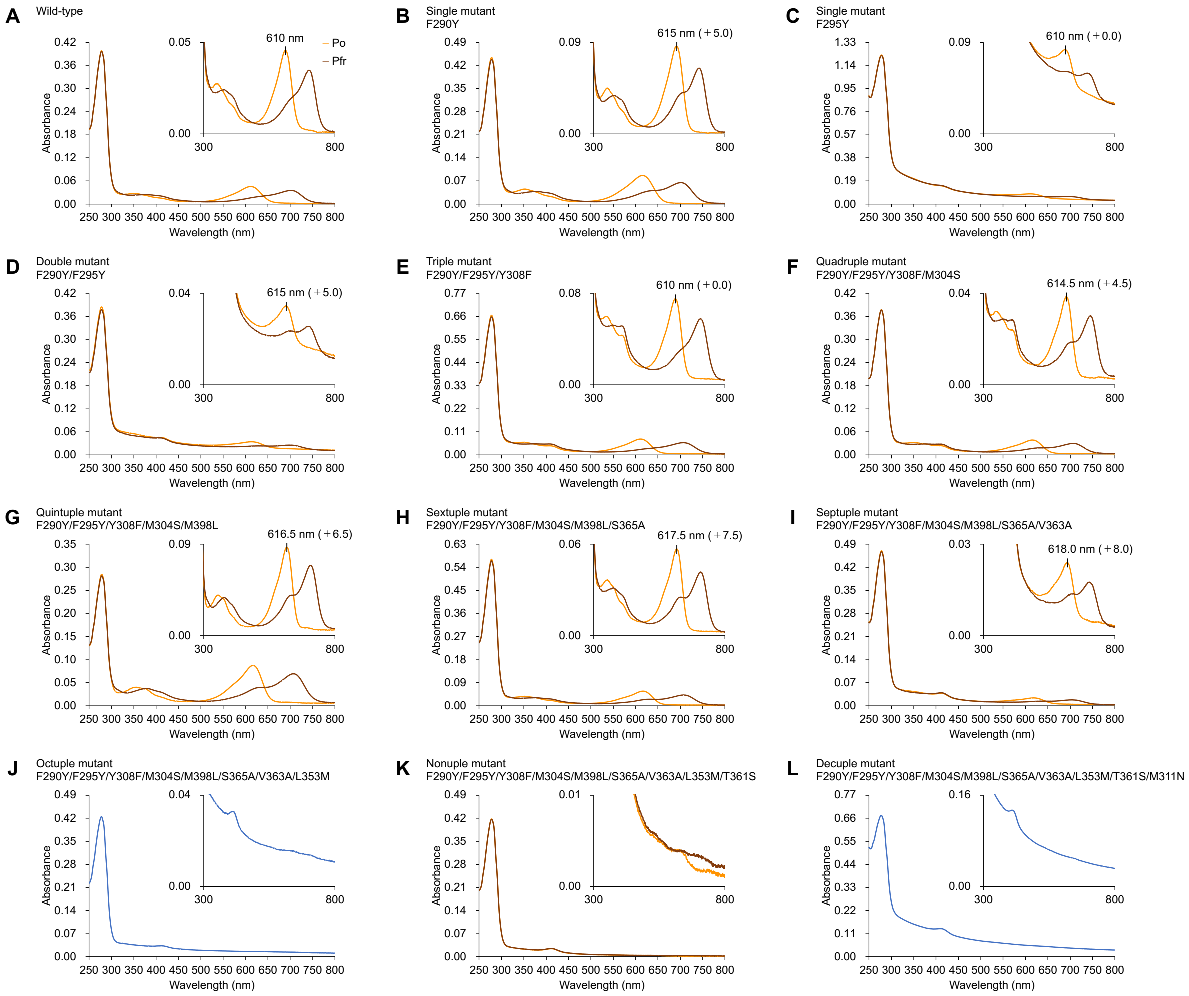

Fig. S5. Absorption spectra of the PCB-binding PpDUC1-N wild-type (A), F290Y variant (B), F295Y variant (C), F290Y/F295Y variant (D), F290Y/F295Y/Y308F variant (E), F290Y/F295Y/Y308F/M304S variant (F), F290Y/F295Y/Y308F/M304S/M398L variant (G), F290Y/F295Y/Y308F/M304S/M398L/S365A variant (H), F290Y/F295Y/Y308F/M304S/M398L/S365A/V363A variant (I), F290Y/F295Y/Y308F/M304S/M398L/S365A/V363A/L353M variant (J), F290Y/F295Y/Y308F/M304S/M398L/S365A/V363A/L353M/T361S variant (K), and F290Y/F295Y/Y308F/M304S/M398L/S365A/V363A/L353M/T361S/M311N variant (L) molecules in the Po (orange) dark-adapted and Pfr (deep red) photoproduct states.

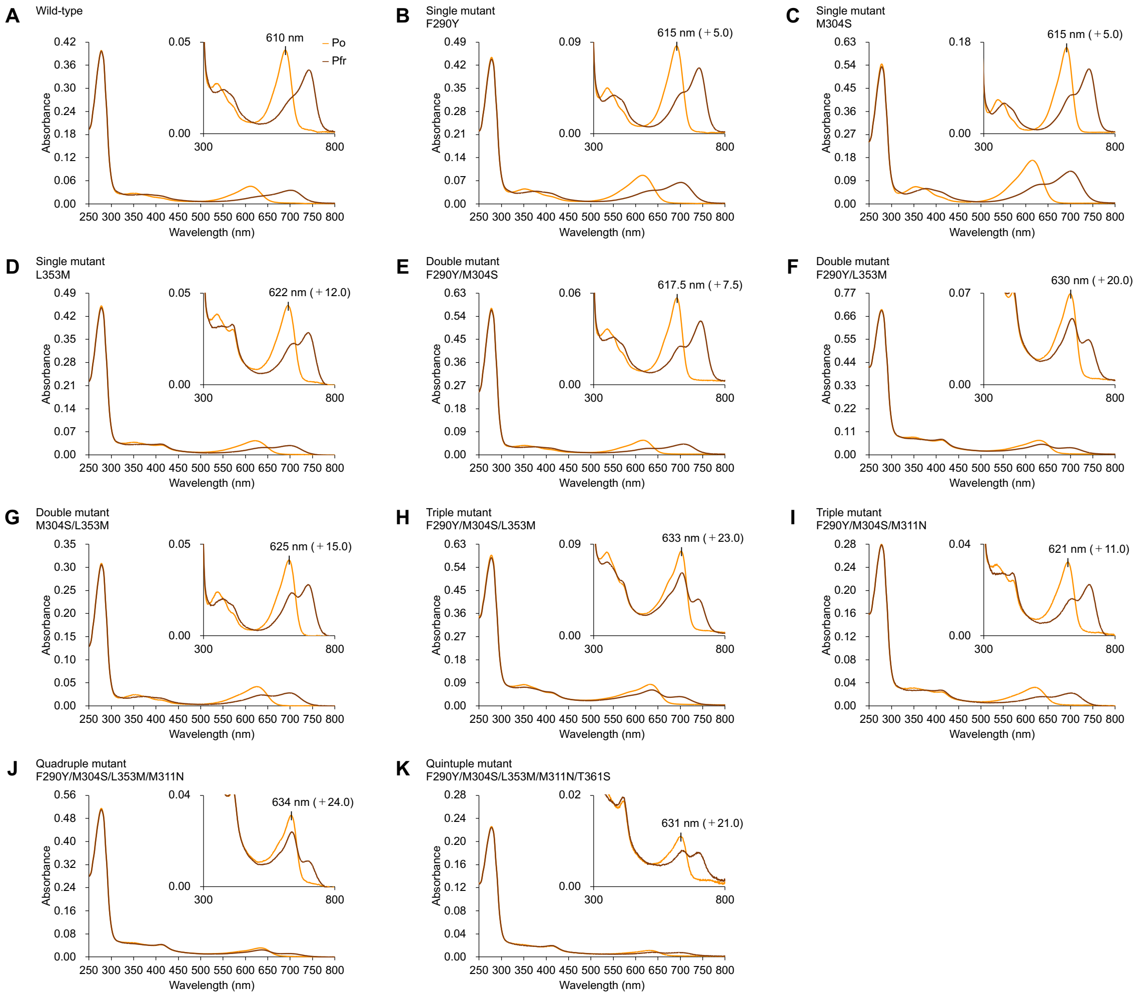

Fig. S6. Absorption spectra of the PCB-binding PpDUC1-N wild-type (A), F290Y variant (B), M304S variant (C), L353M variant (D), F290Y/M304S variant (E), F290Y/L353M variant (F), M304S/L353M variant (G), F290Y/M304S/L353M variant (H), F290Y/M304S/M311N variant (I), F290Y/M304S/L353M/M311N variant (J), and F290Y/M304S/L353M/M311N/T361S variant (K) molecules in the Po (orange) dark-adapted and Pfr (deep red) photoproduct states.

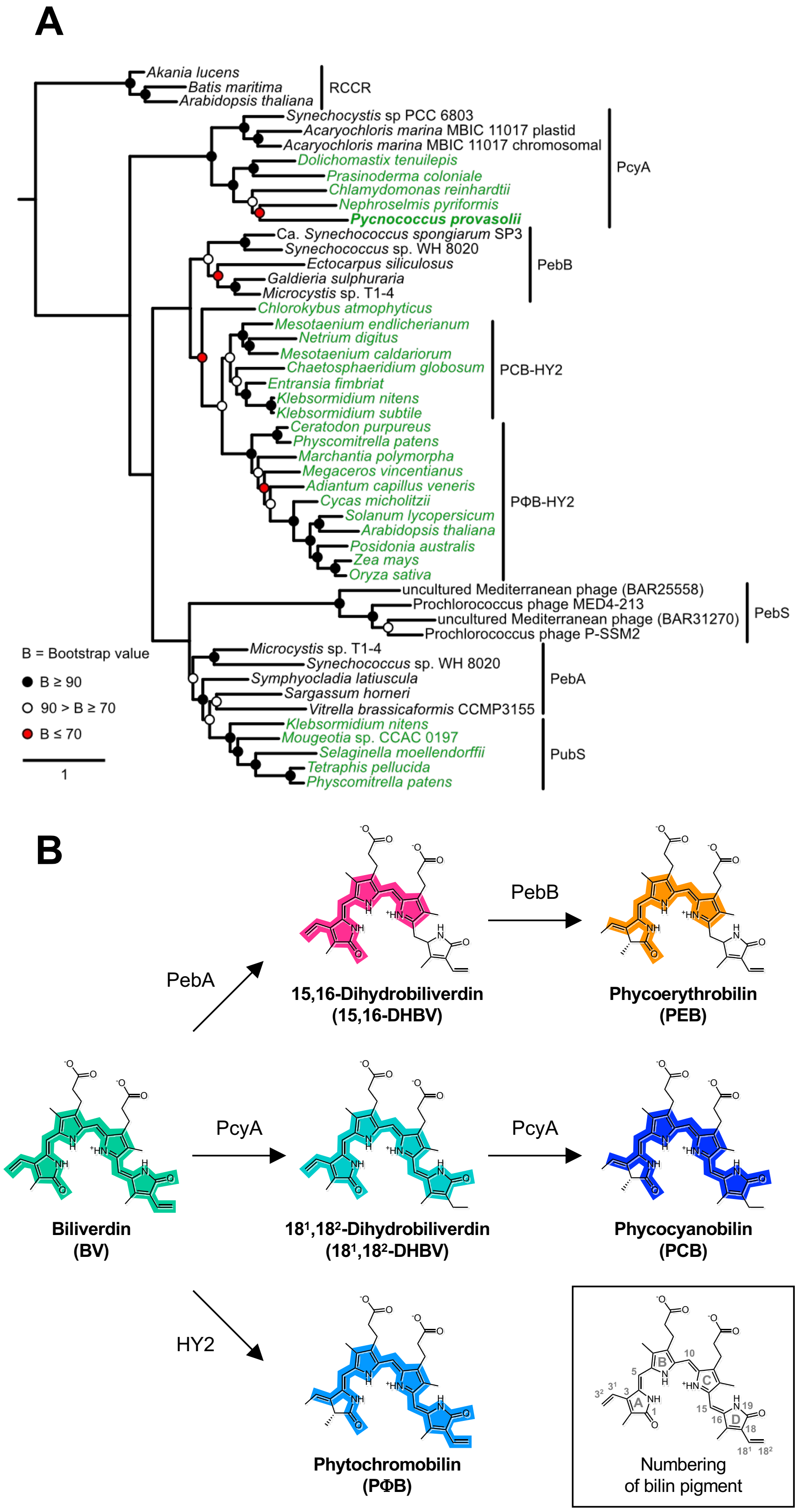

Fig. S7. Classification of FDBR enzymes. (A) Maximum likelihood phylogenetic tree of FDBRs with the RCCR sequences set as an out-group. The green algae and plants are shown in green. The bold one is characterized in this study. (B) Enzyme reaction scheme of the FDBR family.
